# Draft Genome Sequence of *Comamonas thiooxydans* Strain S23^T^ (DSM 17888^T^), a Thiosulfate-Oxidizing Bacterium Isolated from a Sulfur Spring in India

**DOI:** 10.1128/genomeA.00834-16

**Published:** 2016-08-11

**Authors:** Kunwar Digvijay Narayan, Jhasketan Badhai, William B. Whitman, Subrata K. Das

**Affiliations:** a Department of Biotechnology, Institute of Life Sciences, Nalco Square, Bhubaneswar, India; b Department of Microbiology, University of Georgia, Athens, Georgia, USA

## Abstract

The genus *Comamonas* contains species isolated from various environments, such as termite guts, wetlands, activated sludge, soil, humans, and fresh water. Here, we report the draft genome sequence of *Comamonas thiooxydans* strain S23^T^ capable of oxidizing thiosulfate under mixotrophic growth conditions. Based upon draft genome sequencing, the genome is 5.3 Mb and encodes 4,767 proteins. The *Comamonas thiooxydans* whole-genome sequence will help understand the metabolic diversity in sulfur oxidation pathways.

## GENOME ANNOUNCEMENT

*Comamonas thiooxydans* strain S23^T^ (DSM 17888^T^) is a Gram-negative aerobic rod. It belongs to the family *Comamonadaceae* of the class *Betaproteobacteria*. The bacterium was isolated from a sulfur spring located at Athmallik, Orissa, India (1). It is the first reported member of the genus *Comamonas* that is capable of oxidizing thiosulfate under mixotrophic growth conditions (2, 3).

The draft genome of *Comamonas thiooxydans* strain S23^T^ was generated at the Department of Energy (DOE) Joint Genome Institute (JGI) using the Illumina HiSeq 2000 platform (4). An Illumina standard shotgun library was constructed and sequenced using the Illumina HiSeq 2000 platform, which generated 8,941,414 reads totaling 1,350.2 Mb. The filtered Illumina reads were assembled using the Velvet (5), wgsim (https://github.com/lh3/wgsim), and Allpaths-LG (6) tools. The final draft assembly contains 45 contigs in 40 scaffolds, totaling 5.3 Mb, with an input read coverage of 253.1-fold. The largest and *N*_50_ contigs are 832.8 kb and 312.4 kb, respectively, with a G+C content of 62.0%.

The genome was annotated using the JGI Microbial Genome Annotation Pipeline (7). Genes were identified using the Prodigal (8) and GenePRIMP (9) programs. The tRNA, rRNA, and other noncoding RNA genes were identified by searching the genome using the tRNAscan-SE tool (10), rRNA gene models built from SILVA (11) and INFERNAL (http://infernal.janelia.org). The draft genome contains 4,767 coding sequences (CDSs), 68 tRNAs, 11 rRNAs, and 10 other RNAs.

### Accession number(s).

This whole-genome shotgun project has been deposited at DDBJ/EMBL/GenBank under the accession no. LIOM00000000. The version described in this paper is the first version, LIOM01000000.
